# A Gravity‐driven Microfluidic Platform for High‐throughput Culture and Inflammatory Stimulation of Human Articular Chondrocytes

**DOI:** 10.1002/cpz1.70489

**Published:** 2026-10-22

**Authors:** Aldeliane M. da Silva, Meriem Lamghari, Hoang‐Tuan Nguyen, Jere Kettunen, Sebastien Mosser, Prateek Singh, Ali Mobasheri, Gabriela S. Lorite, Priscila Campioni Rodrigues

**Affiliations:** ^1^ Microelectronics Research Group University of Oulu Pentti Kaiteran Katu 1 Oulu Finland; ^2^ i3S ‐ Instituto de Inovação e Investigação em Saúde Universidade do Porto Rua Alfredo Allen, 208 Porto Portugal; ^3^ Proteins.1 Espoo Finland; ^4^ Finnadvance Helsinki Finland; ^5^ Research Unit of Health Sciences and Technology, Faculty of Medicine University of Oulu Oulu Finland; ^6^ Department of Personalized Medicine State Research Institute Centre for Innovative Medicine Vilnius Lithuania; ^7^ Department of Joint Surgery First Affiliated Hospital of Sun Yat‐sen University Guangzhou China; ^8^ Faculté de Médecine Université de Liège Liège Belgium; ^9^ Department for Health Sciences, Medicine and Research, Center for Regenerative Medicine, Faculty of Health and Medicine University for Continuing Education Krems Krems Austria; ^10^ Department of Orthopedic Surgery Maastricht University Medical Center Maastricht The Netherlands

**Keywords:** chondrocytes, dynamic cell culture, gravity‐driven flow, inflammation, microfluidics

## Abstract

Articular cartilage is a load‐bearing connective tissue whose structural integrity depends on a highly specialized extracellular matrix and the activity of resident chondrocytes. In vivo, chondrocytes are continuously exposed to mechanical stimuli, including fluid‐induced shear stress associated with synovial fluid movement, which plays a critical role in regulating cell phenotype and matrix homeostasis. Osteoarthritis (OA) is a degenerative joint disease marked by extracellular matrix breakdown, chondrocyte dysfunction, and inflammation. Despite its high prevalence, effective disease‐modifying therapies remain unavailable, in part due to the lack of in vitro models that recapitulate both the biochemical and biomechanical environment of native cartilage. This article describes a pumpless, gravity‐driven microfluidic platform for modelling inflammatory OA‐like conditions under dynamic flow, based on and expanding upon our previously published work. This system is based on gravity‐driven flow generated by a rocker platform, eliminating the need for external pumps and enabling scalable, parallelized operation. Defined microchannel geometries allow spatially resolved exposure of cells to varying shear stress levels within a single device. Human articular chondrocytes are cultured under dynamic conditions and stimulated with interleukin‐1β to induce inflammatory responses. The protocols presented here include preparation and extracellular matrix coating of the microfluidic chips, measurement of flow dynamics under rocker‐induced motion, long‐term dynamic culture of chondrocytes under gravity‐driven flow, and induction of OA‐like inflammatory conditions followed by evaluation of chondrocyte responses. The platform supports assessment of cellular responses, including viability, metabolic activity, expression of major extracellular matrix markers (collagen type II and aggrecan), and secretion of inflammatory mediators. This approach provides a reproducible in vitro model that integrates dynamic cell culture and inflammation while offering a scalable alternative to conventional static cultures and externally actuated organ‐on‐chip systems. The method enables efficient investigation of chondrocyte inflammatory responses and has potential applications in OA disease modelling and drug screening. © 2026 The Author(s). *Current Protocols* published by Wiley Periodicals LLC.

**Basic Protocol 1**: Extracellular matrix coating and operation of a gravity‐driven microfluidic chip for long‐term culture of human articular chondrocytes.

**Support Protocol 1**: Measurement of gravity‐driven flow rate in microfluidic chips under rocker‐induced tilt.

**Basic Protocol 2**: Seeding and long‐term dynamic culture of human articular chondrocytes under gravity‐driven flow.

**Basic Protocol 3**: Induction of osteoarthritis‐like inflammatory conditions using IL‐1β under gravity‐driven flow and analysis of chondrocyte responses.

## INTRODUCTION

Articular cartilage is a specialized connective tissue composed of chondrocytes embedded within a dense extracellular matrix (ECM) rich in collagen type II, proteoglycans, glycosaminoglycans, and water. Chondrocytes are the sole resident cell population and maintain tissue homeostasis through balanced synthesis and degradation of ECM components (Eschweiler et al., 2021; Petitjean et al., 2023). *In vivo*, articular cartilage is exposed to mechanical stimuli, including compressive loading and fluid‐induced shear stress (FSS) generated by synovial fluid movement during joint articulation. These mechanical inputs are known to contribute to the regulation of chondrocyte phenotype and cartilage integrity through mechanosensitive signaling pathways (Jia et al., 2023; Q. Liu et al., 2016).

Osteoarthritis (OA) is a progressive degenerative joint disease characterized by ECM breakdown, chondrocyte dysfunction, and low‐grade inflammation (Carpintero‐Fernández et al., 2022; Hunter et al., 2020). Despite its high global prevalence and associated socioeconomic burden, disease‐modifying therapeutic options remain unavailable, and current clinical management is limited to symptom relief. A contributing factor to the limited translation of candidate therapies is the lack of in vitro models that recapitulate both the biochemical and biomechanical microenvironment of native cartilage tissue (Dou et al., 2023; Manivong et al., 2023). Conventional systems include two‐dimensional monolayer cultures, animal models, and engineered three‐dimensional constructs such as hydrogels and organoids (Foster et al., 2022). Two‐dimensional cultures are widely used because they are reproducible but do not recapitulate native cell‐matrix interactions or physiological mechanical cues. Animal models offer greater biological complexity but are limited by interspecies differences that reduce their translational relevance. These limitations have contributed to the development of microengineered systems, including organ‐on‐chip platforms that integrate microfluidics, three‐dimensional culture, and controlled mechanical stimulation (Ingber, 2022; Tolabi et al., 2023).

Cartilage‐on‐chip systems have commonly implemented externally actuated mechanical stimulation, including pneumatic compression systems or magnetically responsive hydrogel‐based approaches (Lee et al., 2018; H. Liu et al., 2024; Paggi et al., 2022; Peitso et al., 2024). While these systems can reproduce selected mechanical aspects of cartilage physiology, they often require external pumping or actuation equipment, which may limit scalability and parallel experimental operation. In parallel, inflammatory OA‐like conditions are frequently induced using recombinant interleukin‐1β (IL‐1β) in two‐dimensional cultures, spheroids, and selected microfluidic platforms (Conceição et al., 2025; Grenier et al., 2014; Scalzone et al., 2023). However, integrated systems combining controlled mechanical stimulation, inflammatory induction, and scalable experimental design remain limited. FSS generated by synovial fluid flow represents a physiologically relevant mechanical cue in articular cartilage. Lower levels of shear stress have been associated with maintaining chondrocyte viability and ECM synthesis, whereas elevated shear stress has been associated with catabolic responses and ECM degradation (Smith et al., 1995; Zhao et al., 2024). Although shear stress has been widely investigated in other mechanosensitive cell types, its application in scalable microfluidic systems for human articular chondrocytes remains limited.

In this article, a commercially available pumpless microfluidic system platform (Finnadvance) based on gravity‐driven flow generated by controlled rocker tilting is employed. The system operates without external pumps or tubing and incorporates parallel microchannels with defined geometries that generate spatial gradients of FSS within a single device. This configuration enables simultaneous exposure of cells to multiple FSS levels and supports long‐term dynamic culture under continuous flow. The system is adapted for human articular chondrocytes and combined with IL‐1β stimulation to establish an inflammatory, OA‐like environment under controlled shear stress. This article builds on a previously developed microfluidic platform (FinnAdvance) for generating spatially defined FSS gradients in human articular chondrocytes, in which differential cellular responses to coating composition, mechanical stimulation, and IL‐1β stimulation were demonstrated (da Silva et al., 2026).

The system enables quantitative assessment of chondrocyte responses to mechanical and inflammatory stimuli. Typical readouts include cell viability and metabolic activity, ECM production (including collagen type II and aggrecan expression), and secretion of inflammatory mediators such as interleukins and matrix metalloproteinases (MMPs). These parameters enable evaluation of anabolic and catabolic processes under defined mechanical conditions. Compared with conventional static culture systems, this platform enables spatially defined FSS gradients while reducing reagent consumption and improving experimental reproducibility. Compared with externally actuated organ‐on‐chip systems, the pumpless configuration eliminates the need for external pumping hardware, thereby simplifying operation and increasing scalability.

Here, we provide a detailed, reproducible workflow (Fig. 1) to enable implementation of this system for modelling OA‐like inflammatory conditions under dynamic flow. Basic Protocol 1 describes preparation and surface coating of the microfluidic device and the establishment of gravity‐driven flow conditions. Basic Protocol 2 describes seeding and long‐term dynamic culture of human articular chondrocytes under gravity‐driven flow. Basic Protocol 3 describes the induction of OA‐like inflammatory conditions using IL‐1β under dynamic flow, followed by analysis of cellular viability, ECM production, and inflammatory marker secretion.

**Figure 1:**
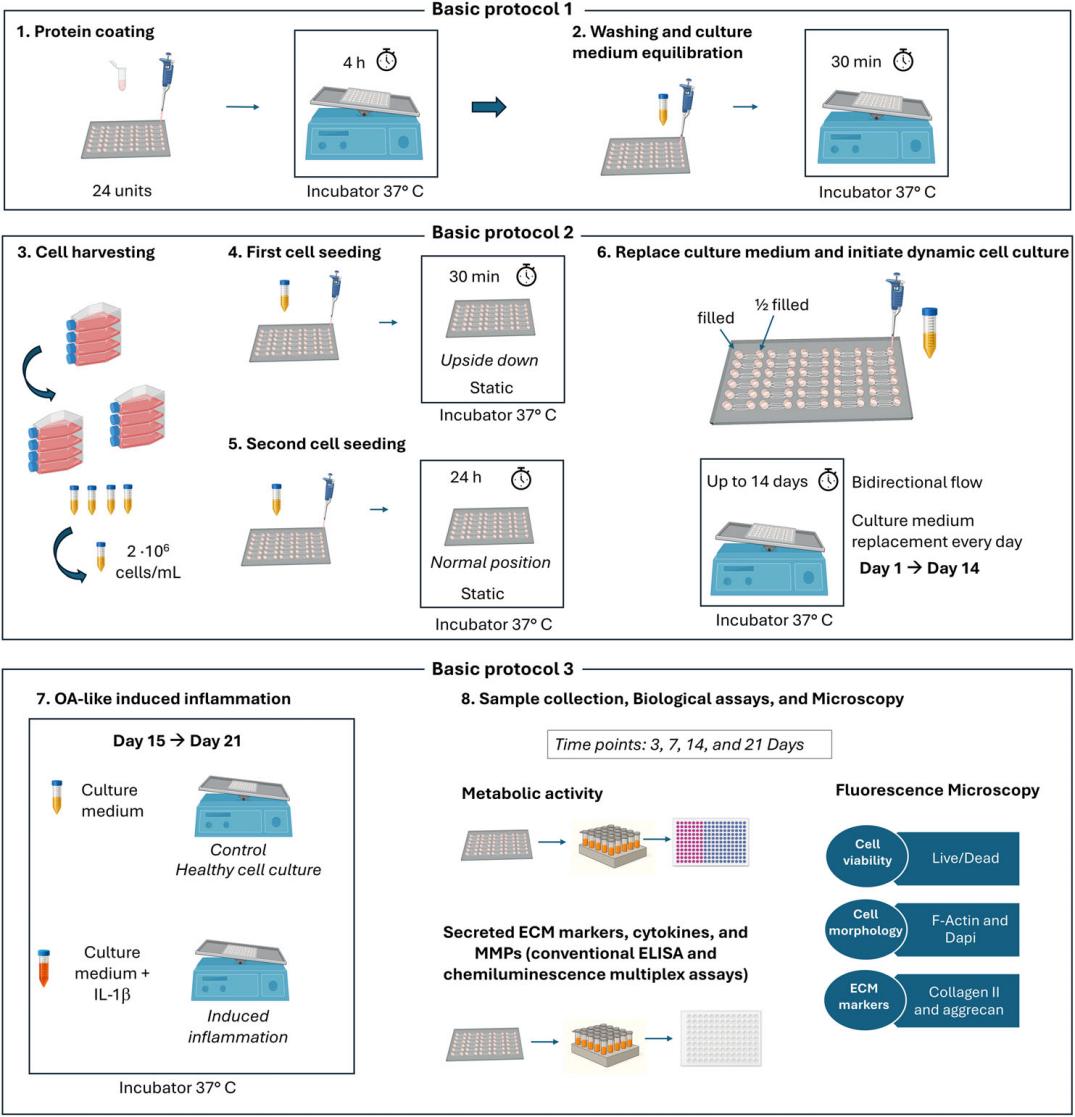
Overview of the experimental workflow for dynamic microfluidic culture of human articular chondrocytes and induction of osteoarthritis‐like inflammation under gravity‐driven flow. Schematic representation of the integrated workflow comprising three basic protocols. Basic Protocol 1: extracellular matrix (ECM) protein coating and equilibration of microfluidic chips. Basic Protocol 2: harvesting, sequential seeding of human articular chondrocytes, and initiation of gravity‐driven bidirectional flow through asymmetric reservoir filling and rocker‐induced tilting, enabling dynamic culture for up to 21 days. Basic Protocol 3: induction of OA‐like inflammation using IL‐1β (days 15–21), followed by downstream analyses at defined time points. Representative readouts include metabolic activity, secretion of ECM components, cytokines, and MMPs, and fluorescence imaging of cell viability, morphology, and ECM marker expression. Note: Some graphical elements were created using BioRender.

*CAUTION*: All procedures involving human chondrocytes must be performed under Biosafety Level 2 (BSL‐2) conditions in a certified Class II biological safety cabinet using appropriate personal protective equipment (laboratory coat, gloves, and protective eyewear). Standard aseptic techniques are required. Biosafety cabinets should be decontaminated before use by UV‐C irradiation, followed by cleaning with 70% (v/v) ethanol. Procedures involving 96% ethanol or other volatile reagents must be performed in a chemical fume hood. UV‐C sterilization must be conducted with appropriate shielding to prevent operator exposure.

*CAUTION*: Care must be taken during all fluid handling steps involving the microfluidic chips, including microchannel loading, cell seeding, and medium exchange, to avoid air bubble formation. Air bubbles can disrupt hydrodynamic flow, alter local FSS profiles, and compromise experimental reproducibility.

*NOTE*: Use of human‐derived cells, including commercially sourced cell lines, must comply with all applicable institutional, national, and international guidelines. Commercial human cell lines used in this protocol do not require ethical approval in most jurisdictions; however, users must confirm local regulatory requirements before use.

## STRATEGIC PLANNING

Before initiating the protocol, users should ensure access to prefabricated 24‐unit gravity‐driven microfluidic chips (Finnadvance or functionally equivalent validated designs), as channel geometry and device architecture influence fluid transport, hydrodynamic behavior, and experimental reproducibility.

Key experimental parameters must then be defined before starting the study. The choice of ECM coating is a critical determinant of initial chondrocyte attachment and subsequent behavior under dynamic flow conditions. Although the platform is compatible with a range of ECM coatings, including fibronectin, collagen type IV, laminin‐521, and mixed formulations, the standard coating used in this protocol is a fibronectin‐collagen type IV mixture. This coating was selected based on previous optimization studies demonstrating improved chondrocyte adhesion, viability, metabolic activity, and long‐term stability under gravity‐driven flow conditions (da Silva et al., 2026). Alternative coatings may be used for comparative studies or protocol optimization but should remain consistent throughout a given experimental series.

The rocker tilt angle is another critical parameter, as it determines the magnitude of flow rate and fluid transport within the microchannels. Previous numerical characterization of the platform demonstrated that changes in rocker tilt angle also influence the predicted FSS distribution within the microchannels (da Silva et al., 2026). For the experiments described here, a tilt angle of ±25° is used as the standard dynamic culture condition. This setting supports long‐term chondrocyte culture by ensuring adequate nutrient delivery and removal of cellular waste products and accumulated secreted factors from the microenvironment. Asymmetric reservoir filling and a fixed dwell time of 300 sec per tilt position should also be maintained to ensure consistent hydrodynamic conditions between experiments.

For OA‐like modelling, inflammatory stimulation is induced using IL‐1β at a concentration of 1 or 10 ng/ml from day 15 to day 21 of culture while maintaining dynamic flow. These conditions were selected to induce a reproducible catabolic response characterized by increased secretion of inflammatory mediators and matrix‐degrading enzymes, together with reduced expression of cartilage‐specific ECM markers. Users interested in studying earlier or more subtle inflammatory responses may consider lower IL‐1β concentrations, as both 1 and 10 ng/ml elicited robust inflammatory and catabolic responses in this system (da Silva et al., 2026). Any modifications to cytokine concentration or exposure duration should be maintained consistently throughout a given experimental series.

Collectively, the selection of ECM coating, flow conditions, and inflammatory stimulation parameters determines initial cell adhesion, long‐term culture stability, and the magnitude of mechanobiological and inflammatory responses. Once established, these conditions should be maintained consistently across experiments to ensure reproducibility and facilitate comparison between experimental groups.

## Basic Protocol 1 ECM COATING AND OPERATION OF A GRAVITY‐DRIVEN MICROFLUIDIC CHIP FOR LONG‐TERM CULTURE OF HUMAN ARTICULAR CHONDROCYTES

This protocol describes the preparation and operation of commercially available gravity‐driven microfluidic chips for culturing human articular chondrocytes under gravity‐driven flow conditions. The system consists of polydimethylsiloxane (PDMS)‐based microfluidic chips (Finnadvance) containing multiple parallel microchannels with progressively varying dimensions. Each culture unit contains four parallel rectangular microchannels corresponding to three channel regions: region I (1500 × 200 × 200 µm), region II (1500 × 150 × 150 µm), and region III (1500 × 100 × 100 µm) (Fig. 2). Bidirectional gravity‐driven flow is generated by asymmetric reservoir loading combined with rocker‐induced tilting, eliminating the need for external pumps or tubing. Proper chip handling, ECM coating, and hydrostatic loading are essential to establish reproducible flow conditions and stable cell adhesion before dynamic culture.

**Figure 2:**
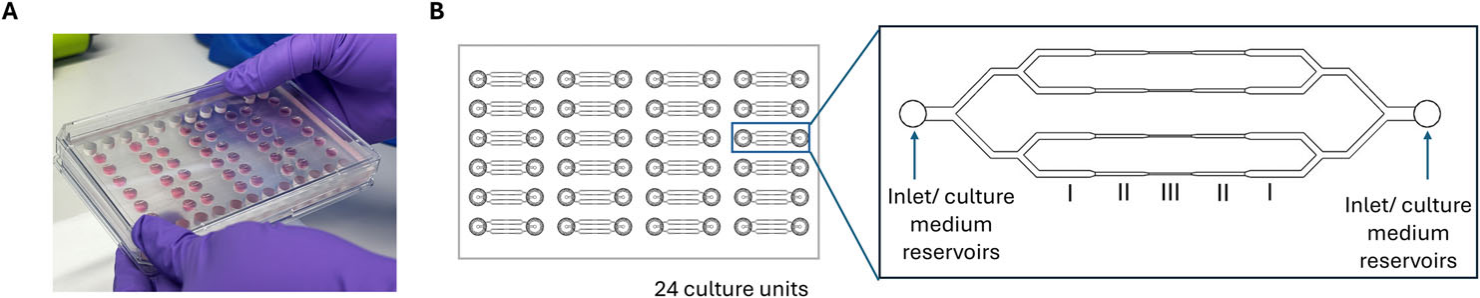
Microfluidic platform and chip layout for dynamic chondrocyte culture under gravity‐driven flow. (A) Photograph of the microfluidic chip with reservoirs filled with culture medium, illustrating the assembled device used for experiments. (B) Schematic representation of the microfluidic chip layout showing an array of 24 culture units. Each unit comprises two culture medium reservoirs connected by four microchannels with varying width and height, subdivided into regions I–III to generate spatial gradients of fluid shear stress under gravity‐driven flow conditions.

### Materials

Dulbecco's phosphate‐buffered saline (DPBS) (Gibco, cat. no. 14190144)
Ethanol (96%)
Human Fibronectin (Corning, cat. no. 356008)
Human Collagen type IV (Sigma‐Aldrich, cat. no. 5533)
Chondrocyte growth medium (HC Growth Medium, Cell Applications, cat. no. 411‐500)

PDMS microfluidic chips (24‐unit gravity‐driven design, FinnAdvance)
Rocker platform (AKITA rocker, FinnAdvance or equivalent)
UV‐C sterilization system
Micropipettes and sterile filtered tips
CO_2_ incubator (37°C, 5% CO_2_)
Biosafety cabinet (Class II)
Stereomicroscope

#### Chip inspection and sterilization

1 Fill the microchannels with sterile DPBS and inspect microfluidic chips under a stereomicroscope to confirm channel integrity and absence of debris or visible leakage.
  Ensure no particulate contamination that could obstruct microchannels.
2 (Additional step, perform only if chips are not pre‐sterilized) Sterilize chips by rinsing 3× with 96% ethanol followed by UV‐C exposure until completely dry.

#### ECM coating

3 Prepare an ECM coating solution in DPBS containing fibronectin (100 µg/ml) and collagen type IV (400 µg/ml).
4 Fill microchannels by pipetting approximately 70 µl coating solution into a single inlet until all channels are completely filled. Inject the coating solution slowly and gently through the inlet, avoiding contact between the pipette tip and the bottom film of the chip, as illustrated in Fig. 3.
  Improper pipette handling may damage the bottom film of the microfluidic chip, resulting in leakage and rendering the affected culture unit unsuitable for further use.

**Figure 3:**
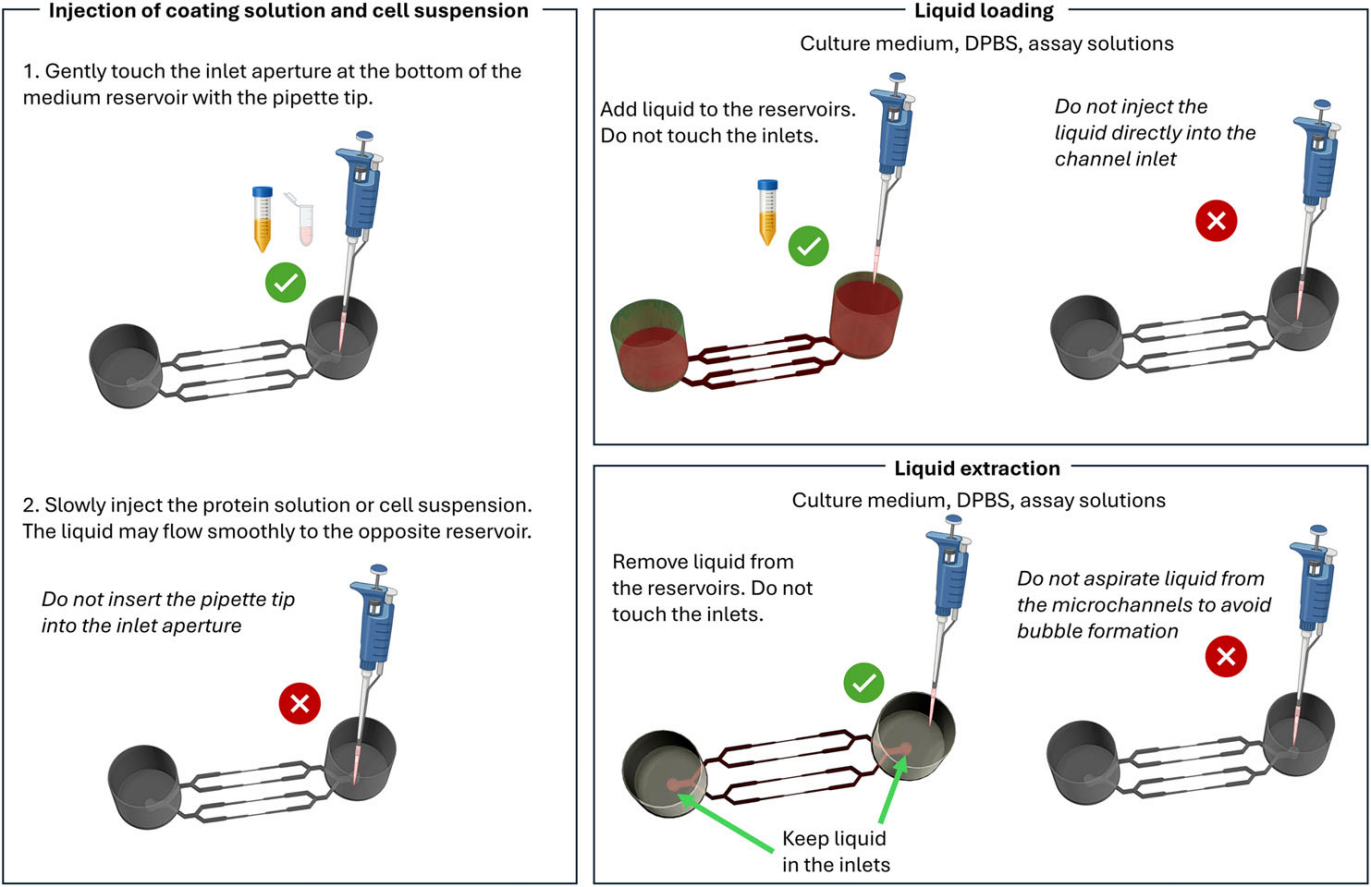
Proper pipette handling for liquid injection, loading, and extraction in microfluidic culture units. Schematic representation of recommended pipette handling techniques for introducing and removing liquids in microfluidic chips. Injection of coating solution and cell suspension: The pipette tip should gently contact the inlet aperture at the bottom of the reservoir without insertion into the channel inlet, followed by slow injection to allow smooth flow through the microchannels toward the opposite reservoir. Direct insertion of the pipette tip into the inlet should be avoided to prevent damage to the channel structure and leakage. Liquid loading (culture medium, DPBS, assay solutions): Add liquids directly to the reservoirs without contacting the inlet apertures. Direct injections into the channel inlet should be avoided to prevent flow disruption and the introduction of air bubbles. Liquid extraction: Liquids should be removed from the reservoirs without contacting the inlet apertures to ensure that the microchannels remain filled. Aspiration from the microchannels should be avoided to prevent bubble formation and loss of fluid continuity within the system. Correct handling is indicated by green check symbols, while incorrect procedures are indicated by red crosses.

5 Place chips on a rocker platform and incubate at 37°C and 5% CO_2_ for 4 hr under oscillation (±25° tilt angle, 1 rpm, 5 min dwell time at each maximum tilt position).
6 Remove coating solution and gently rinse the microchannels by adding DPBS to the medium reservoirs and carefully tilting the chip back and forth to promote flow through the channels. Do not inject DPBS directly into the channel inlets. Add DPBS only to the reservoirs, avoiding contact between the pipette tip and the inlet apertures, as shown in Fig. 3. After rinsing, add pre‐warmed chondrocyte growth medium and incubate for 30 min before cell loading. If a longer delay is expected before cell seeding, maintain the channels filled with sterile DPBS to prevent drying.
  Avoid complete drying of the microchannels after coating, as this may increase nonspecific protein adsorption and reduce coating uniformity.

At completion of this protocol, the microfluidic chips are uniformly coated with ECM proteins and prepared for cell seeding. The system is ready for long‐term dynamic culture of human articular chondrocytes under gravity‐driven flow conditions as described in Basic Protocol 2.

## Support Protocol 1 MEASUREMENT OF GRAVITY‐DRIVEN FLOW RATE IN MICROFLUIDIC CHIPS UNDER ROCKER‐INDUCED TILT

This protocol describes the experimental determination of gravity‐driven flow rates in the microfluidic chip under rocker‐induced bidirectional flow conditions. The method quantifies transient fluid transport during dwell periods at defined tilt angles and validates the system's hydrodynamic behavior. Flow rates are determined by gravimetric measurement of medium transferred between reservoirs over time.

### Materials

Chondrocyte culture medium (HC Growth Medium, Cell Applications, cat. no. 411‐500)
DPBS (sterile, Gibco, cat. no. 14190144)
Human Fibronectin (Corning, cat. no. 356008)
Human Collagen type IV (Sigma‐Aldrich, cat. no. C5533)

Prefabricated gravity‐driven microfluidic chips (24‐unit design, FinnAdvance or equivalent)
Rocker platform (AKITA rocker, FinnAdvance or equivalent)
CO_2_ incubator (37°C, 5% CO_2_)
Analytical balance (precision ± 0.1 mg)
Sterile pipettes and filtered tips
15‐ and 50‐ml sterile tubes
Sterile glass vials or chip inlet/outlet collection containers

#### Chip preparation and equilibration

1 Pre‐coat microchannels with a mixture of fibronectin (100 µg/ml) and collagen type IV (400 µg/ml) in DPBS for 4 hr at 37°C.
  This step ensures consistent surface conditions for flow characterization.
2 Incubate chips with chondrocyte culture medium for 2 hr at 37°C and 5% CO_2_ prior to flow measurement.

#### Reservoir loading and initial setup

3 Remove culture medium from the reservoirs and keep the channels filled to avoid bubble formation.
4 Refill one inlet reservoir completely with culture medium (100 µl).
5 Refill the opposing reservoir to half of its total volume (50 µl) to establish an initial hydrostatic imbalance. This asymmetry is required to generate reproducible gravity‐driven flow.

#### Flow measurement under rocker‐induced tilt

6 Place the microfluidic chip on a rocker platform at room temperature set to reach tilt angles of ±25°, rotation rate of 1 rpm, and dwell time of 300 sec at each maximum tilt position ±25°.
7 Define *t*_0_ = 0 sec as the moment the rocker reaches the 25° tilt position, marking the onset of the dwell period.
8 At *t*_0_ = 0 sec, collect the volume present in the outlet reservoir (*V*₀), which represents the initial reservoir volume (50 µl) plus the volume transferred from the onset of tilting until the beginning of the dwell period. Similarly, collect the outlet reservoir volumes at the following time points: *t*_1_ = 60 sec, *t*_2_ = 120 sec, *t*_3_ = 180 sec, and *t*_4_ = 240 sec. For each sampling time point, use a separate microfluidic culture unit and collect the contents of the outlet reservoir into a pre‐weighed container (e.g., a glass vial or microcentrifuge tube) for gravimetric analysis.
  The collection should be performed rapidly and consistently to minimize experimental variation.
9 Determine the collected volume by gravimetric measurement using an analytical balance. Assume a medium density of approximately 1 g/ml for conversion of mass to volume.
10 Repeat the measurement using five independent microfluidic units for each sampling time point to ensure reproducibility.

#### Data processing and flow rate calculation

11 Define the measured volumes at *t*_0_ = 0 sec, *t*_1_ = 60 sec, *t*_2_ = 120 sec, *t*_3_ = 180 sec, and *t*_4_ = 240 sec as outlet reservoir volumes (*V*_0_, *V*_1_, *V*_2_, *V*_3_, and *V*_4_, respectively), where each volume corresponds to the total volume present in the outlet reservoir at the specified time point.
12 Determine the average flow rate for each sampling interval as: *Q_i_* (*t*) = Δ*V_i_*/Δt, where Δ*t* = 60 sec and Δ*V_i_* = *V_i_* − *V_i_* ₋₁, which corresponds to the transferred volume during the 60 sec sampling interval.
13 Plot flow rate as a function of time to characterize transient decay during the dwell period.

This procedure yields time‐resolved flow rate profiles under gravity‐driven bidirectional flow conditions, enabling quantitative validation of hydrodynamic behavior in gravity‐driven microfluidic platforms. The workflow is summarized in Fig. 4.

**Figure 4:**
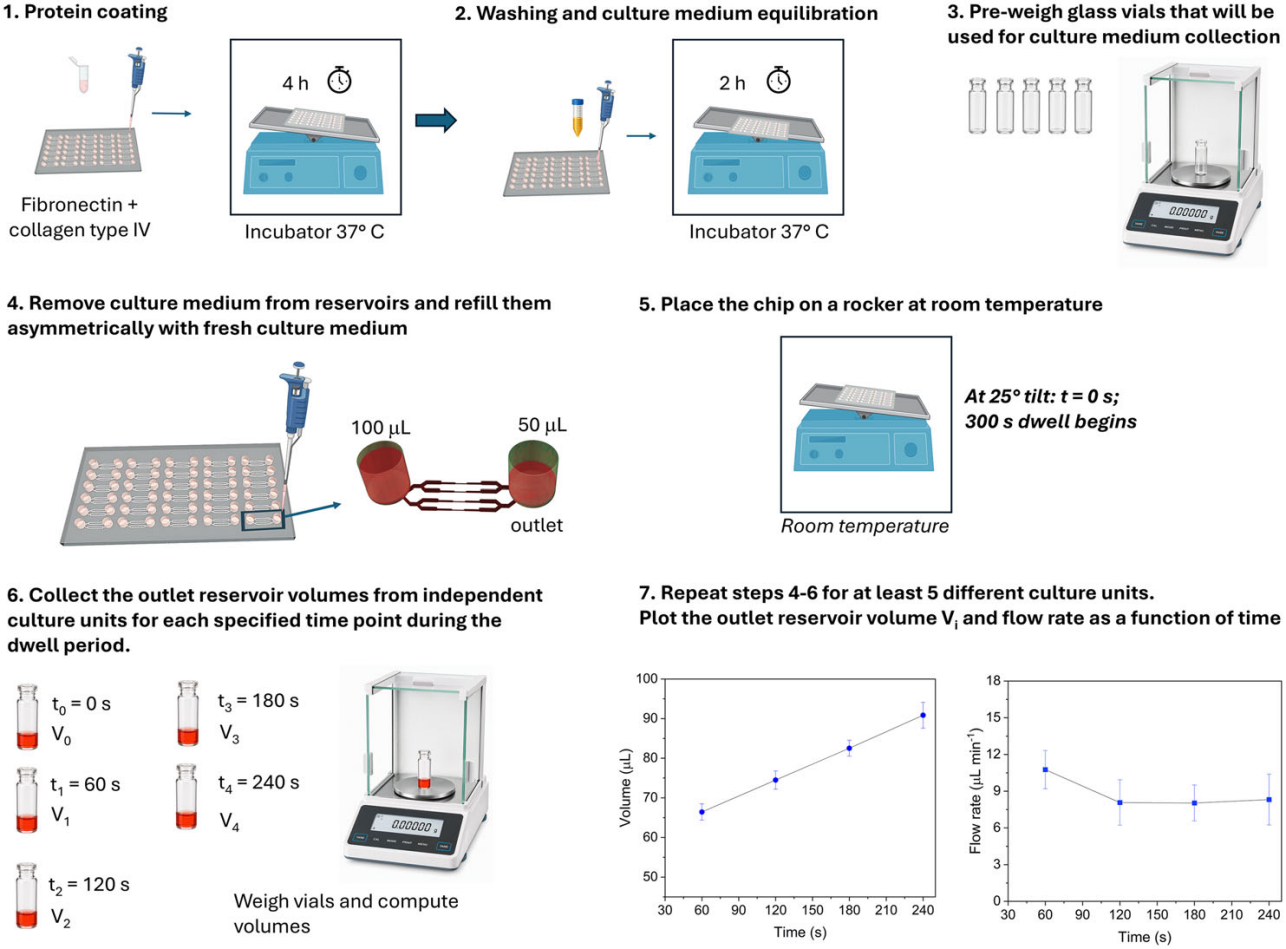
Workflow for experimental determination of gravity‐driven flow rate in microfluidic chips under rocker‐induced tilt. Schematic overview of the experimental procedure used to measure flow rates during the dwell period at a defined tilt angle (±25°). (1–2) Microfluidic chips are coated with extracellular matrix (ECM) proteins (fibronectin‐collagen type IV mixture) and equilibrated with culture medium under incubator conditions. (3) Collection vials are pre‐weighed using an analytical balance prior to flow measurements. (4) Reservoirs are asymmetrically filled (e.g., 100 µl vs. 50 µl) to establish a hydrostatic pressure difference driving fluid flow between reservoirs. (5) The chip is placed on a rocker platform at room temperature and operated under defined conditions (±25° tilt, 1 rpm, 300 sec dwell period). The dwell period begins at the maximum tilt position (25°) and is defined at *t* = 0 sec. (6) During the 300‐sec dwell period, the cumulative transferred volume to the initially lower‐volume reservoir is collected at defined time points (e.g., 60, 120, 180, and 240 sec) using pre‐weighed vials. For each time point, independent microfluidic units are used. The collected volumes are determined by gravimetric measurement. (7) Measurements are repeated across five independent culture units per sampling time point. Transferred volumes and flow rates are calculated as Δ*V_i_* = *V_i_* − *V_i_*_−1_ and *Q_i_* = Δ*V_i_*/Δ*t*, and plotted as a function of time. Note: Graphs are reproduced from da Silva et al. (2026) licensed under CC BY 3.0.

## Basic Protocol 2 SEEDING AND LONG‐TERM DYNAMIC CULTURE OF HUMAN ARTICULAR CHONDROCYTES UNDER GRAVITY‐DRIVEN FLOW

This protocol describes seeding and long‐term culture of human articular chondrocytes in a gravity‐driven microfluidic chip. Cells are introduced into ECM‐coated microchannels and cultured under rocker‐induced bidirectional flow to generate spatial FSS gradients. Proper cell loading, initial static adhesion, and controlled initiation of dynamic flow are critical for achieving stable long‐term culture and reproducible mechanobiological responses.

### Materials

Human articular chondrocytes (cryopreserved, commercial source, Cell Applications, cat. no. 402‐05)
Chondrocyte growth medium (HC Growth Medium, Cell Applications, cat. no. 411‐500)
DPBS (sterile, Gibco, cat. no. 14190144)
Trypsin EDTA (Sigma‐Aldrich, T3924)
Defined trypsin inhibitor (Cascade Biologics, Gibco, cat. no. R007100)
Trypan blue

Pre‐coated PDMS microfluidic chips (from Basic Protocol 1)
15‐ and 50‐ml sterile tubes
Rocker platform (AKITA or equivalent)
CO_2_ incubator (37°C, 5% CO_2_)
Centrifuge
T75 and T175 cell culture flasks
Biosafety cabinet (Class II)
Micropipettes (10‐, 200‐, and 1000‐µL range) and sterile filtered tips
Hemocytometer or automated cell counter
Microscope

#### Cell preparation and counting

1 Thaw and expand human articular chondrocytes according to supplier instructions until the desired cell number is obtained.
  Cells between passages 2 and 5 are recommended for reproducible culture performance.
2 Maintain cells under standard culture conditions and avoid over‐confluence prior to cell seeding.
3 Harvest chondrocytes using trypsin‐EDTA according to standard culture procedures (typically 5 min at 37°C), followed by neutralization using trypsin inhibitor‐containing medium. Collect cells by centrifugation (220 × *g*, room temperature, 5 min) and resuspend in chondrocyte growth medium.
4 Determine viable cell concentration using trypan blue exclusion and a hemocytometer or automated cell counter.
  Only suspensions with ≥90% viability should be used for microfluidic seeding to ensure reproducible adhesion and long‐term stability.
5 Adjust cell density to 2 × 10⁶ cells/ml in pre‐warmed chondrocyte growth medium.
  Maintain a homogeneous single‐cell suspension by gentle pipetting; avoid foaming or aggregation.

#### Microfluidic chip preparation (pre‐equilibrated system)

6 Verify ECM‐coated chips from Basic Protocol 1 are equilibrated with chondrocyte growth medium prior to seeding.
7 Remove medium from chip reservoirs while maintaining filled microchannels to prevent air entry and bubble formation.

#### Cell seeding and distribution

8 Seed 35 µL of cell suspension into one inlet of each culture unit and gently inject the suspension through the inlet to fill the microchannels, as illustrated in Fig. 3.
  During injection, avoid contact between the pipette tip and the bottom film of the chip, as this may damage the device and cause leakage.
9 Inspect the microchannels under a microscope, if available, to confirm uniform filling and the absence of air bubbles or cell aggregates.
  Air bubbles disrupt flow patterns and FSS distribution and should be eliminated before proceeding.
10 Invert the chips to promote cell attachment to the upper microchannel surface and improve subsequent cell distribution under dynamic flow conditions. Incubate the chips inverted for 30 min at 37°C and 5% CO_2_.
11 Return the chips to the upright orientation and add an additional 35 µl cell suspension through the inlet to promote uniform cell distribution. Gently tilt chips manually back and forth to facilitate redistribution of the newly added cell suspension.
  Perform manual tilting gently to avoid introducing air bubbles or dislodging already attached cells.

#### Static adhesion phase

12 Incubate chips under static conditions for 24 hr at 37°C and 5% CO_2_.
  This step ensures stable cell adhesion before flow exposure and reduces detachment during initial shear‐stress loading.

#### Initiation of gravity‐driven dynamic culture

13 Remove culture medium from the reservoirs and establish a hydrostatic imbalance by filling one reservoir completely with 100 µl pre‐warmed culture medium and the opposite reservoir to half volume with 50 µl medium.
14 Place the chips on a rocker platform set to ±25° tilt, 1 rpm rotation rate, and a dwell time of 300 sec at each maximum tilt position.
15 Define the initiation of rocker‐induced bidirectional flow as *t* = 0 hr of dynamic culture.
16 Maintain continuous bidirectional gravity‐driven flow throughout the culture period.

#### Long‐term dynamic culture maintenance

17 Replace culture medium daily while maintaining the same asymmetric reservoir filling (100 µl vs. 50 µl) configuration to preserve consistent hydrodynamic conditions and FSS profiles.
  During medium exchange, ensure that the microchannels remain filled with liquid to prevent air bubble formation and flow disruption.
18 Maintain dynamic culture for 14 days under gravity‐driven flow conditions. This period constitutes the pre‐stimulation culture phase of the model. Following completion of the 14‐day culture period, proceed to Basic Protocol 3 for induction of OA‐like inflammatory conditions using IL‐1β under dynamic flow.

## Basic Protocol 3 INDUCTION OF OA‐LIKE INFLAMMATORY CONDITIONS USING IL‐1β UNDER GRAVITY‐DRIVEN FLOW AND ANALYSIS OF CHONDROCYTE RESPONSES

This protocol is performed after Basic Protocol 2, which establishes long‐term chondrocyte cultures under gravity‐driven flow. IL‐1β is applied to dynamically cultured cells to reproduce inflammatory conditions while maintaining defined FSS gradients. The protocol includes cytokine stimulation, continued dynamic culture, assessment of cell morphology, viability, and metabolic activity, immunofluorescence analysis of ECM markers, and quantification of inflammatory mediators released into the culture medium.

### Materials

Chondrocyte growth medium (HC Growth Medium, Cell Applications, cat. no. 411‐500)
DPBS (sterile, Gibco, cat. no. 14190144)
Recombinant IL‐1β (R&D Systems, cat. no. 201‐LB‐025) (separately at −20°C)
Live/Dead Viability/Cytotoxicity Kit containing calcein‐AM and ethidium homodimer 1 (EthD‐1) (Invitrogen, cat. no. LS224)
PrestoBlue Cell Viability Reagent (Invitrogen, cat. no. A13261)
4% paraformaldehyde (PFA) in DPBS (Thermo Scientific, cat. no. J61899.AP)
Normal donkey serum
Bovine serum albumin (BSA)
Triton X‐100
Tetramethylrhodamine (TRITC)‐phalloidin staining (EMD Millipore/Merck, cat. no. 90228)
Mouse anti‐Collagen II antibody (NB600‐844, Novus Biologicals)
Goat anti‐Aggrecan antibody (AF1220, R&D Systems)
Rabbit anti‐Mouse IgG (H+L), FITC‐conjugated secondary antibody (NB7543, Novus Biologicals)
Donkey anti‐Goat IgG (H+L), Northern Lights 557 (NL557)‐conjugated secondary antibody (NL001, R&D Systems)
4′,6‐diamidino‐2‐phenylindole (DAPI) Solution (Novus Biologicals, Bio‐Techne, cat. no. NBP2‐31156)
Q‐Plex Human MMP 6 Plex kit (340949HU, Quansys Biosciences)
Q‐Plex Human Custom Cytokine Plex kit (107749GR, Quansys Biosciences)
Human collagen type II alpha 1 ELISA kit (BSKH63293, Bioss Antibodies)
Pre‐established chondrocyte cultures generated using Basic Protocol 2
Rocker platform (AKITA rocker, FinnAdvance or equivalent)
CO_2_ incubator maintained at 37°C and 5% CO_2_
Class II biosafety cabinet
Wide‐field fluorescence microscope (e.g., Leica DM4B or equivalent) equipped with blue/green and red/green excitation/emission filter sets.
Wide‐field fluorescence microscope equipped for z‐stack imaging (e.g., Leica DM6B Thunder Microscope or equivalent) with UV/blue, blue/green, and red/green excitation/emission filter sets. Alternatively, a confocal microscope may be used.
Black 384‐well plates
Transparent 96‐well plates
Microplate reader
Microplate shaker (capable of reaching 500 rpm)
Q‐View Imager LS (Quansys Biosciences)
Centrifuge
Refrigerated microcentrifuge
Single‐channel micropipettes (10, 200, and 1000 µl range) and sterile filtered tips
Multichannel micropipette (5 to 300 µl range) and sterile filtered tips
15‐ and 50‐ml tubes
Sterile microcentrifuge tubes (0.6 ml and 2.0 ml)

#### Preparation of IL‐1β stimulation medium

1 Prepare IL‐1β working solutions in chondrocyte growth medium at final concentrations of 1 ng/ml and 10 ng/ml.
2 Prepare cytokine‐free control medium using the same basal culture medium formulation.
3 Pre‐warm all media to 37°C immediately before use.

#### Inflammatory stimulation under gravity‐driven flow

4 Remove existing medium from the reservoirs and replace it with either IL‐1β‐containing medium or cytokine‐free control medium. Maintain identical loading volumes and reservoir filling configurations across all experimental groups.
  Keep the microchannels filled with liquid during medium exchange to prevent air bubble formation.
5 Place microfluidic chips on the rocker platform using the same operating parameters established in Basic Protocol 2 (±25° tilt angle, 1 rpm, 300 sec dwell time).
6 Maintain continuous bidirectional gravity‐driven flow throughout the stimulation period.
7 Continue IL‐1β exposure for 7 days (culture days 15–21).
8 Replace medium daily with freshly prepared IL‐1β‐containing or control medium while maintaining identical rocker settings and reservoir filling conditions across all experimental groups.

#### Live/dead viability staining

9 Prepare Live/Dead staining solution containing 0.25 µM calcein‐AM and 0.25 µM EthD‐1 in pre‐warmed chondrocyte culture medium.
10 Remove culture medium from the reservoirs while ensuring that the microchannels remain filled to prevent air entry into the system.
11 Add staining solution to the reservoirs and incubate chips on the rocker platform at 37°C and 5% CO_2_ for 30 min, protected from light.
12 Remove the staining solution and gently rinse samples by adding warm DPBS to the reservoirs and carefully tilting the chip back and forth to promote flow through the channels. Do not inject DPBS directly into the inlets. Add pre‐warmed culture medium to the reservoirs.
13 Acquire fluorescence images from regions I, II, and III using a wide‐field fluorescence microscope (e.g., Leica DM4B or equivalent) equipped with appropriate filters for calcein‐AM (green; excitation/emission maxima: 494/517 nm) and EthD‐1 (red; excitation/emission maxima: 528/617 nm). Use a low‐magnification objective (e.g., 10×) to capture larger microchannel areas.
  Alternatively, a confocal microscope may be used when higher‐resolution imaging or optical sectioning is required.
14 Acquire multiple representative fields of view from each region (I–III) for all biological replicates to ensure robust assessment of cell viability and spatial variability across microchannels.

#### Stain F‐actin (TRITC‐phalloidin) and cell nuclei (DAPI)

15 Remove culture medium from the reservoirs and gently rinse microchannels with DPBS.
16 Fix cells by incubating with 4% paraformaldehyde in DPBS for 15 min at room temperature.
17 Wash samples three times with DPBS (150 µl per culture unit) to remove residual fixative.
18 Incubate samples overnight at 4°C in blocking/permeabilization buffer consisting of DPBS containing 10% normal donkey serum, 1% BSA, and 0.1% Triton X‐100.
19 Prepare a staining solution containing TRITC‐phalloidin (reconstituted to 60 µg/ml stock concentration; 1:800 dilution) in blocking/permeabilization buffer.
20 Incubate samples with TRITC‐phalloidin staining solution for 2 hr at room temperature, protected from light.
21 Wash samples three times with DPBS to remove unbound stain.
22 Counterstain nuclei with DAPI (1 mg/ml stock solution; 1:2000 dilution in DPBS) for 5 min at room temperature.
23 Perform a final wash with DPBS and maintain samples in DPBS prior to imaging.
  All washing and incubation steps were performed on a rocker platform (e.g., AKITA) to ensure uniform reagent distribution within the microchannels.
24 Acquire fluorescence images using a wide‐field fluorescence microscope (e.g., Leica DM6B Thunder) equipped with a motorized stage for z‐stack acquisition.
  Alternatively, a confocal microscope may be used to acquire z‐stack images with improved optical sectioning.
25 Perform tile scanning and z‐stack acquisition to capture large channel areas from bottom to top. Apply computational clearing (Thunder processing) to reduce out‐of‐focus light.
26 Image F‐actin using TRITC filter settings (excitation: 532–558 nm; emission: 572–648 nm) and nuclei using DAPI filter settings (excitation: 382–408 nm; emission: 435–485 nm) with a 40× objective.

#### Immunofluorescence staining for ECM markers

27 Remove culture medium from reservoirs and rinse the microchannels with DPBS.
28 Fix cultures by incubating with 4% paraformaldehyde in DPBS for 15 min at room temperature.
29 Wash samples twice with DPBS for 5 min each.
30 Incubate samples for 1 hr at room temperature in blocking/permeabilization buffer (DPBS containing 10% normal donkey serum, 1% BSA, and 0.1% Triton X‐100).
31 Prepare primary antibody solution in blocking buffer: Mouse monoclonal anti‐Collagen II antibody (1:200; NB600‐844, Novus Biologicals), Goat polyclonal anti‐Aggrecan antibody (5 µg/ml; AF1220, R&D Systems)
32 Add primary antibody solution to each culture unit and incubate overnight at 4°C on a rocker platform.
33 Wash samples four times with DPBS for 2 min each.
34 Prepare secondary antibody solution in blocking buffer: Rabbit anti‐Mouse IgG (H+L), FITC‐conjugated secondary antibody (1:200; NB7543, Novus Biologicals) for detection of collagen type II; and Donkey anti‐Goat IgG (H+L), NL557‐conjugated secondary antibody (1:200; NL001, R&D Systems) for detection of aggrecan.
35 Incubate samples for 2 hr at room temperature, protected from light.
36 Wash samples four times with DPBS.
37 Counterstain nuclei with DAPI (1 mg/ml stock solution; 1:2000 dilution in DPBS) for 5 min.
38 Perform final DPBS wash and fill reservoirs with DPBS prior to imaging.
39 Acquire fluorescence z‐stack images using a Leica DM6B Thunder wide‐field microscope (or equivalent) equipped with appropriate fluorescence filter sets:
  a. DAPI (blue, cell nuclei): excitation 358 nm, emission 461 nm
  b. FITC (green, collagen type II): excitation 495 nm, emission 519 nm
  c. NL557 (red, aggrecan): excitation 557 nm, emission 574 nm
    Images may be acquired sequentially to minimize spectral overlap. Use a 20× objective for image acquisition. Alternatively, z‐stack images may be acquired using a confocal microscope to improve optical sectioning and resolution.
40 Generate z‐projections for comparative analysis between experimental groups.
  Unless otherwise stated, all washing and incubation steps were performed on a rocker platform set to a ±25° tilt angle, 1 rpm, and a 1 min dwell time to ensure uniform reagent distribution within the microchannels. For each unit, 150 µl solution was used, distributed asymmetrically across the reservoirs (e.g., 100 µl in one reservoir and 50 µl in the other) to facilitate gravity‐driven flow through the microchannels.

#### Z‐stack processing and image preparation

41 Export z‐stack images acquired with a Leica DM6B Thunder wide‐field microscope after computational clearing as multichannel grayscale TIFF files.
42 Import image stacks into Fiji (ImageJ).
43 Assign lookup tables (LUTs) to each fluorescence channel for visualization:
  a. Collagen type II → green LUT
  b. Aggrecan → red LUT
  c. DAPI → blue LUT
    LUT assignment is used for visualization purposes only and does not alter the underlying pixel intensity values.
44 Adjust brightness and contrast as needed to improve visualization of cellular structures and facilitate region‐of‐interest (ROI) selection.
  Do not click the “Apply” button when adjusting brightness and contrast, as this will permanently modify pixel intensity values and compromise quantitative analysis.
45 Verify correct channel assignment and alignment across the z‐stack.
46 Generate z‐projections (e.g., maximum intensity projection) for visualization if required. Representative image processing steps are shown in Fig. 5, including screenshots of Fiji (ImageJ) illustrating:
  a. Z‐stack visualization with merged fluorescence channels
  b. Z‐projection (maximum intensity projection) with merged channels
  c. Individual z‐stacks for each separate channel
  d. The Fiji (ImageJ) toolbar interface and the Brightness and Contrast (B&C) adjustment window.
    The displayed z‐stack images correspond to a single slice (typically the first slice) within the stack. Navigation through additional slices is performed using scroll bar or arrow controls within the Fiji (ImageJ) interface.

**Figure 5:**
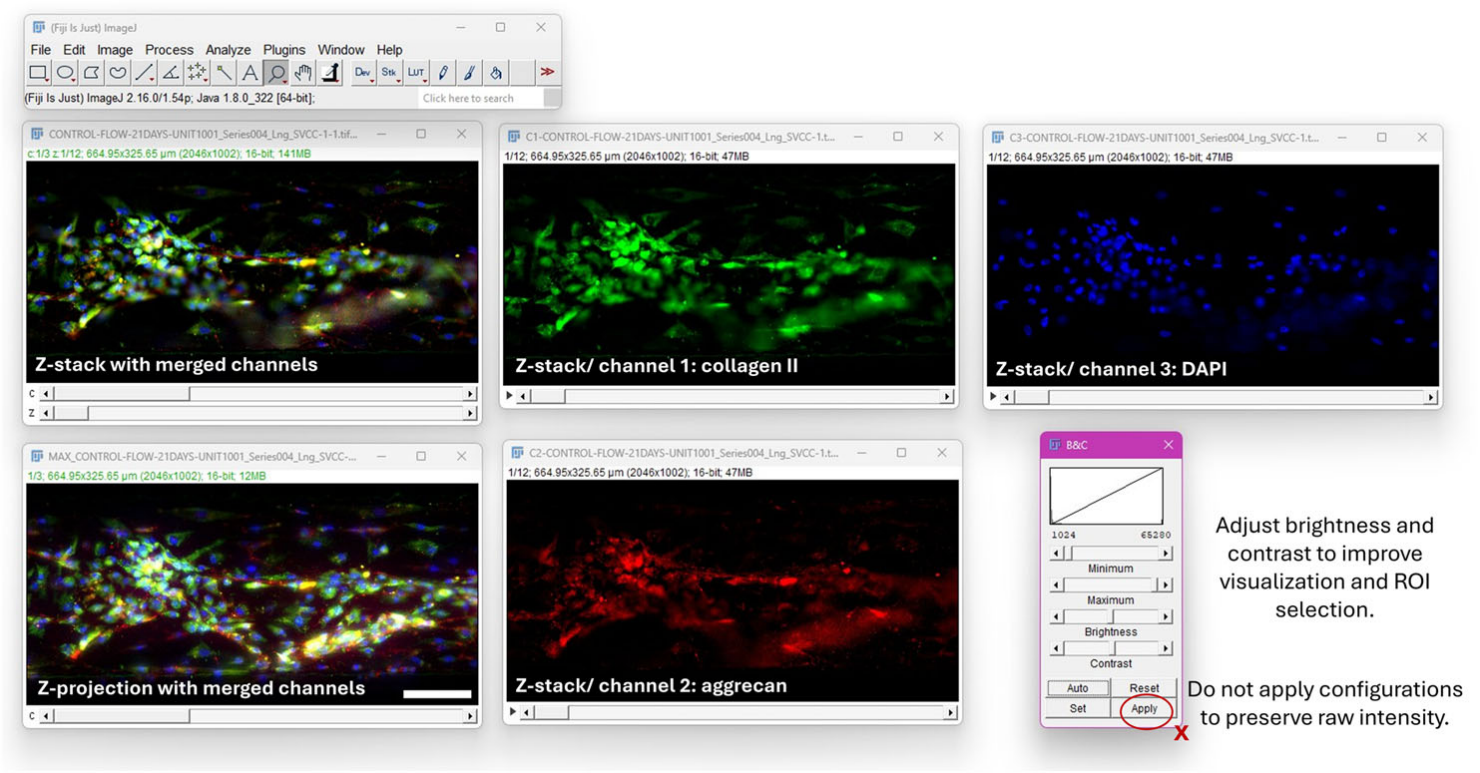
Image processing workflow for z‐stack analysis and fluorescence quantification in Fiji (ImageJ). Screenshots illustrate key steps in image processing and preparation for extracellular matrix (ECM) marker quantification. Panels show representative Fiji (ImageJ) interfaces for: visualization of z‐stack images with merged fluorescence channels (collagen type II, green; aggrecan, red; DAPI, blue); generated z‐projection images with merged channels (Scale bar = 100 µm); individual z‐stacks for each fluorescence channel separately; Fiji (ImageJ) toolbar; and the Brightness & Contrast (B&C) adjustment window. Displayed z‐stack images correspond to a single optical slice (typically the first slice) within the stack. Navigation through the full z‐stack is performed using the scroll bar or arrow controls in the Fiji (ImageJ) interface. Lookup tables (LUTs) are assigned to each channel for visualization purposes only and do not modify the underlying grayscale intensity values. Brightness and contrast may be adjusted to facilitate visualization and accurate ROI selection. However, the “Apply” function should not be used, as it permanently alters pixel intensity values and compromises quantitative fluorescence analysis.

#### Selection of in‐focus cells and ROIs

47 Identify z‐slices corresponding to the bottom region of the microchannel (typically the first 2–3 slices) where cells are most clearly in focus. Representative workflow steps are illustrated in Fig. 6.

**Figure 6.**
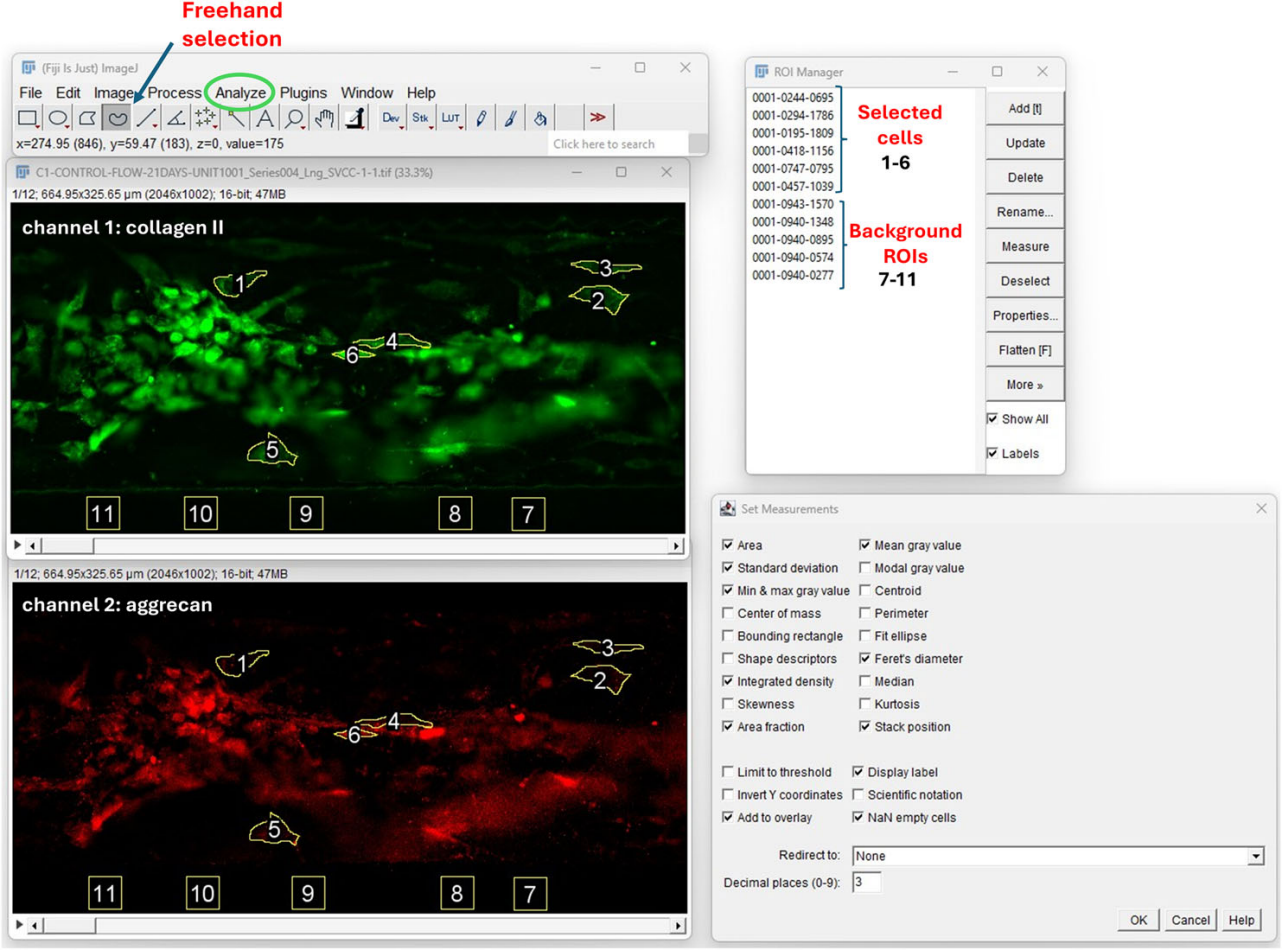
Image processing workflow for ROI selection and fluorescence quantification in Fiji (ImageJ). Screenshots illustrating key steps for ROI selection and fluorescence quantification in Fiji (ImageJ). Representative images show individual fluorescence channels (collagen type II, green; aggrecan, red) from z‐stack data. Displayed images correspond to the first slice of the z‐stack; navigation through slices is performed using the scroll bar or arrow controls in the Fiji (ImageJ) interface. The ROI Manager interface (Analyze → Tools → ROI Manager) was used to define measurement regions. Individual cells were manually selected in the first channel using the freehand selection tool and assigned as ROIs (yellow outlines, labeled 1‐6), then added to the ROI Manager. Additional background ROIs (n = 5) were defined outside the microchannels using square selections and added to the same ROI set. ROIs were subsequently overlaid onto the second channel using the “Show all” function to ensure consistent measurement across channels. Measurement parameters were defined in the Set Measurements menu (Analyze → Set Measurements), and fluorescence intensity values (gray values), integrated density, and area were obtained by selecting each channel and applying the “Measure” function in the ROI Manager.

48 Manually select individual cells in the first fluorescence channel (collagen type II) using ROI tools (e.g., freehand or polygon selection). Add each selected ROI to the ROI Manager (Analyze → Tools → ROI Manager) using the “Add” function. Select cells based on the following criteria:
  a. Cells exhibit sharp, well‐defined morphology (in focus)
  b. Cells are spatially isolated (no overlapping with neighboring cells)
  c. Cells are not part of aggregates or microtissue‐like clusters
    Exclude out‐of‐focus cells and clustered regions, as these may introduce signal contamination from overlapping fluorescence and lead to overestimation of marker expression. Due to residual out‐of‐focus signal inherent to wide‐field imaging, fluorescence quantification was limited to manually selected, in‐focus cells to ensure accurate measurement of marker expression. Automated segmentation approaches can be applied to confocal imaging data, enabling improved optical sectioning and cell boundary detection.

#### Background measurements

49 Define at least five background ROIs using square selections in regions outside the microchannels and add them to the ROI Manager.
50 Ensure that background ROIs are placed in areas free of cells and debris and correspond to the same imaging conditions as the cell ROIs.

#### Fluorescence quantification

51 Apply the defined ROIs across all fluorescence channels by selecting the corresponding channel and enabling the “Show All” function in the ROI Manager.
52 Open the Set Measurements dialog (Analyze → Set Measurements) and select appropriate parameters, including:
  a. Area
  b. Integrated density
  c. Mean gray value
53 Measure fluorescence intensity for each ROI by selecting the corresponding channel and clicking “Measure” in the ROI Manager.
54 Calculate corrected total cell fluorescence (CTCF) for each marker using the formula:
CTCF=Integrateddensity−(Cellarea×Meanbackgroundfluorescence),
  where the mean background fluorescence is calculated from the average of the background ROIs.
55 Repeat measurements across all experimental groups using identical analysis settings.
  Apply consistent ROI selection criteria and use the same number of single‐cell measurements for each experimental condition (e.g., control and IL‐1β–stimulated samples) to ensure comparability.
56 Present representative images and quantitative results (e.g., mean CTCF ± SD, bar plots or box plots). Perform statistical analysis as appropriate.

#### Metabolic activity assay

57 Prepare PrestoBlue working solution consisting of 10% reagent in pre‐warmed chondrocyte culture medium.
58 Remove medium from reservoirs and gently rinse channels with warm DPBS.
59 Add 150 µl PrestoBlue working solution to each culture unit.
60 Incubate chips on the rocker platform for 1 hr at 37°C and 5% CO_2_, protected from light.
61 Collect assay solution from reservoirs into individual sterile microcentrifuge tubes, mix thoroughly, and transfer 40 µl per well into black 384‐well plates.
62 Measure fluorescence using excitation/emission wavelengths of 535 nm/615 nm with a microplate reader.
63 Normalize fluorescence values to blank microfluidic chips containing medium only.

#### Collection and preparation of conditioned medium for ELISA analysis

64 Collect approximately 150 µl conditioned medium from chip reservoirs on day 21 into individual sterile microcentrifuge tubes. Representative workflow steps are shown in Fig. 7A.

**Figure 7:**
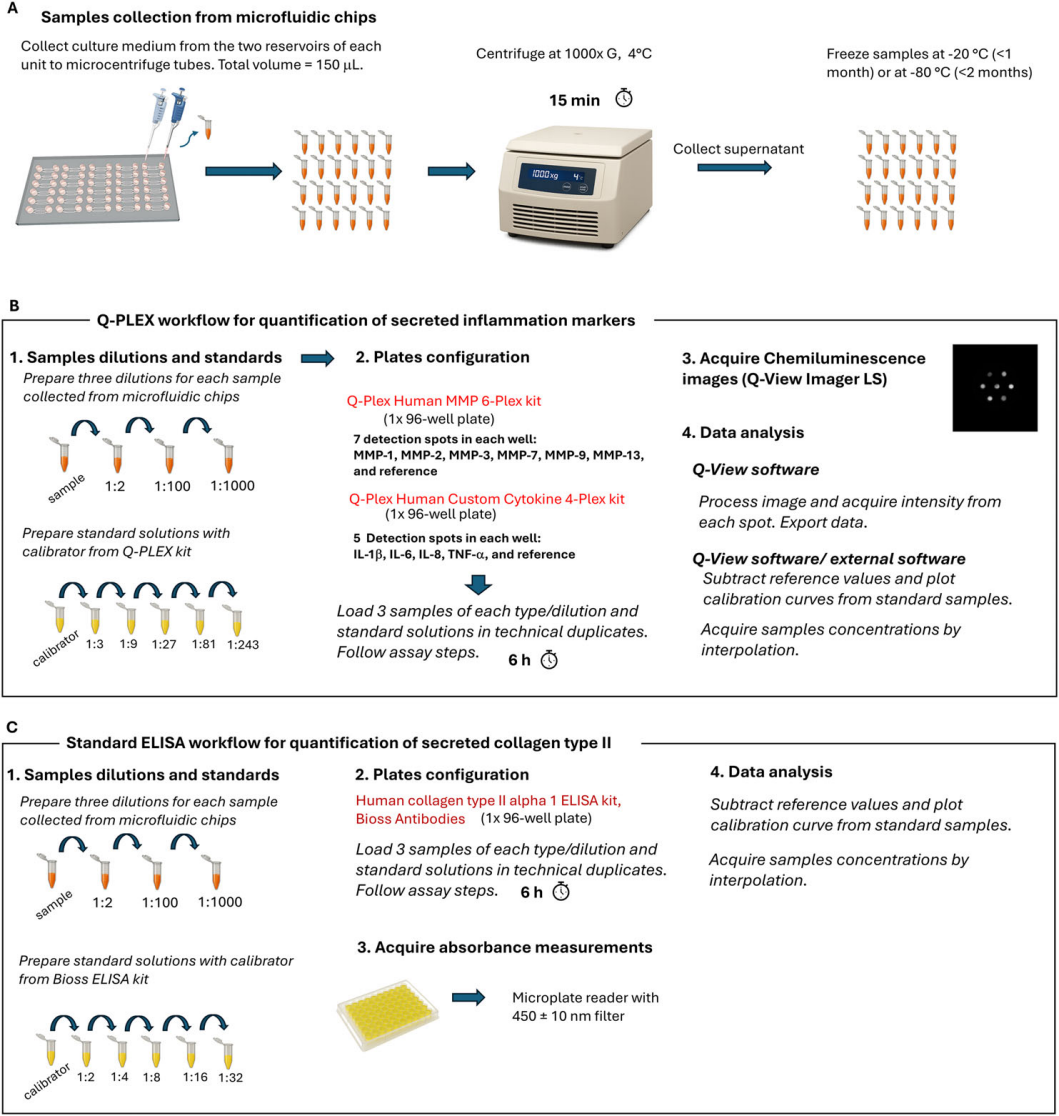
Workflow for ELISA‐based quantification of secreted inflammatory and extracellular matrix (ECM) markers. (A) Collection of culture medium (∼150 µL per unit) from microfluidic chips, followed by centrifugation (1000 × *g*, 4°C, 15 min), supernatant recovery, and storage at −20°C or −80°C. (B) Multiplex ELISA (Q‑PLEX) workflow for quantification of inflammatory mediators (MMPs and cytokines), including sample dilution, plate loading, chemiluminescence detection, and data analysis using calibration curves. (C) Standard ELISA workflow for quantification of collagen type II fragments, including sample dilution, absorbance measurement (450 ± 10 nm), and concentration determination by interpolation.

65 Centrifuge samples to remove cellular debris and particulates using a refrigerated microcentrifuge (1000 × *g*, 4°C, 15 min).
  Sample quality: Proper removal of debris is essential to prevent assay interference. Use a refrigerated microcentrifuge to avoid analyte denaturation.
66 Transfer supernatant to a fresh tube without disturbing the pellet.
67 Store samples at 4°C if used on the same day, at −20°C (up to 1 month) or at −80°C (up to 2 months) for long‐term storage. Avoid repeated freeze‐thaw cycles.
  Repeated freeze‐thaw cycles can degrade proteins and affect quantification accuracy.
68 On the day of ELISA analysis, thaw samples on ice (if frozen) and mix gently.
69 Prepare serial dilutions of each sample (e.g., 1:2, 1:100, and 1:1000) in the appropriate assay diluent, as control and IL‐1β–stimulated samples may contain analytes across different concentration ranges.
  Use consistent dilution factors across replicates to facilitate comparison between conditions. Selecting appropriate dilution factors is critical to ensure measurements fall within the detection range of the standard curves.
  IL‐1β stimulation typically increases secretion of inflammatory mediators and may alter ECM‐related markers such as collagen type II.

#### Multiplex ELISA for MMPs and Cytokines (Q‐Plex)

70 Perform multiplex ELISA assays using Q‐Plex kits (Q‐Plex Human MMP 6 Plex kit and Q‐Plex Human Custom Cytokine Plex kit, Quansys Biosciences) according to the manufacturer's instructions. Representative workflow steps are shown in Fig. 7B.
  Use of Q‐Plex kits requires access to a Q‐View Imager LS for data acquisition.
71 Add standards, controls, and diluted samples to the plate in technical duplicates. Use at least three biological replicates for each sample condition collected from microfluidic chips.
  Including both technical duplicates and biological replicates improves data robustness and measurement accuracy.
72 Acquire chemiluminescent signals using Q‐View Imager LS.
73 Generate standard calibration curves and calculate analyte concentrations using Q‐View software.
  Alternatively, raw chemiluminescence intensity data for each marker can be exported and analyzed using external software by generating calibration curves, applying an appropriate curve fit (e.g., four‐parameter logistic regression), and calculating sample concentrations.
74 Plot average data from all biological replicates for each condition. Express data as concentration (e.g., pg/ml or ng/ml).
  Multiplex ELISA assays efficiently measure multiple analytes from limited samples.
  TNF‐α levels may fall below detection limits depending on the model's system and should be interpreted cautiously.

#### Single‐Analyte ELISA for Collagen type II degradation fragments

75 Perform collagen type II ELISA using the human collagen type II alpha 1 ELISA kit according to the manufacturer's instructions (Bioss Antibodies). Representative workflow steps are shown in Fig. 7C.
76 Load standards, controls, and diluted samples in technical duplicates.
77 Complete all incubation, washing, and detection steps as specified.
78 Measure absorbance using a microplate reader at the appropriate wavelength (e.g., 450 nm).
79 Generate standard calibration curves, apply an appropriate curve fit (e.g., four‐parameter logistic regression), and determine sample concentrations by interpolation.
80 Plot average data from all biological replicates for each condition. Express data as concentration (e.g., pg/ml or ng/ml).

This protocol establishes a dynamically perfused, OA‐like inflammatory microenvironment in human articular chondrocytes under spatially defined FSS gradients. Expected outcomes include disruption of microtissue‐like aggregates, altered metabolic activity, reduced ECM protein expression, and increased secretion of inflammatory mediators and matrix‐degrading enzymes in IL‑1β‐stimulated cultures. Representative results illustrating cell morphology (F‐actin/DAPI staining), cell viability (Live/Dead assay), ECM marker expression (collagen type II and aggrecan immunostaining), metabolic activity (PrestoBlue assay), and secretion of inflammatory markers (matrix metalloproteinases and interleukins) are presented in Fig. 8.

**Figure 8:**
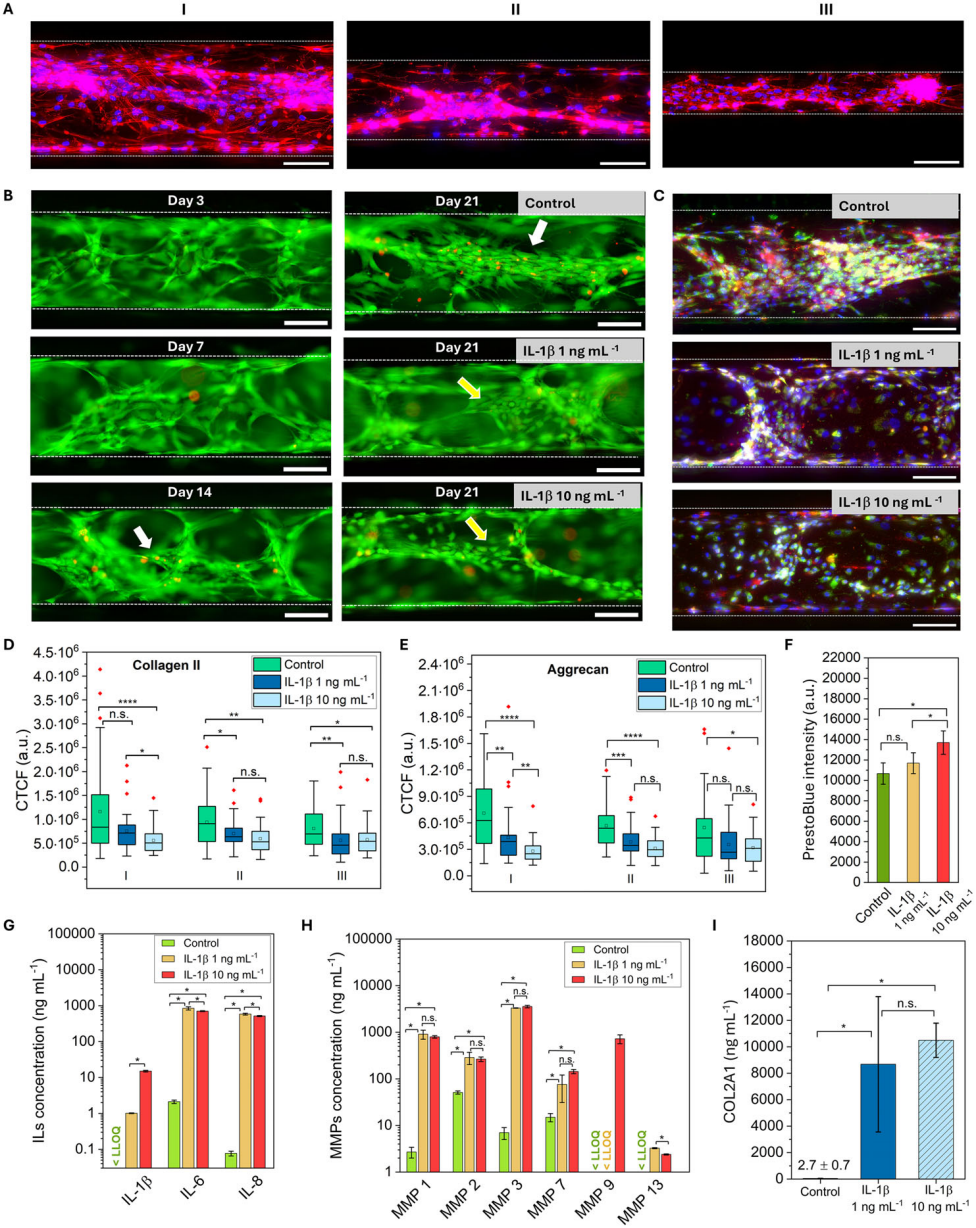
Expected cellular responses of human articular chondrocytes under dynamic culture and IL‑1β‐induced inflammatory conditions. Representative results illustrate chondrocyte morphology, viability, extracellular matrix (ECM) expression, metabolic activity, and inflammatory responses under gravity‐driven flow conditions. (A) Representative z‐projection fluorescence images of chondrocytes cultured for 21 days in microchannels coated with a fibronectin–collagen type IV mixture. Cells form aggregates and microtissue‐like structures across regions I–III corresponding to increasing fluid shear stress levels. Cells are stained for F‐actin (red) and nuclei (DAPI, blue). (B) Representative Live/Dead fluorescence images showing time‐dependent viability and proliferation of chondrocytes cultured under dynamic conditions on days 3, 7, 14, and 21 (control), compared with IL‑1β‐stimulated cultures on day 21 (1 and 10 ng/ml, applied from day 15 to day 21). Control cultures show progressive formation of compact microtissue‐like aggregates (white arrows), whereas IL‑1β stimulation results in disrupted aggregates (yellow arrows). Green: calcein‐AM (live cells); red: ethidium homodimer‐1 (dead cells). (C) Representative z‐projection immunofluorescence images of ECM marker expression in region I after 21 days of culture, comparing control and IL‑1β‐stimulated samples. Images show merged fluorescence channels for collagen type II (green), aggrecan (red), and nuclei (DAPI, blue). (D–E) Quantification of corrected total cell fluorescence (CTCF) for collagen type II (D) and aggrecan (E) across regions I–III for control and IL‑1β‐stimulated cultures at day 21 (n = 44 cells per group). (F) Cell metabolic activity measured using a PrestoBlue assay for control and IL‑1β‐stimulated samples at day 21 (*n* = 5 independent samples per group). (G–H) Quantification of inflammatory mediators in culture supernatant collected at day 21 using multiplex ELISA, including matrix metalloproteinases (MMP‐1, MMP‐2, MMP‐3, MMP‐7, MMP‐9, MMP‐13) and cytokines (IL‑1β, IL‑6, IL‑8) (*n* = 3 independent samples per group). Values below the lower limit of quantification are indicated as <LLOQ. (I) Quantification of collagen type II fragments released into the culture medium at day 21, measured by ELISA (*n* = 3 independent samples per group). All fluorescence images include a 100 µm scale bar, and white dashed lines indicate microchannel boundaries. Statistical analysis was performed using the Kruskal–Wallis test with Dunn's post hoc comparison (*p < 0.05, **p < 0.01, ***p < 0.001, ****p < 0.0001; n.s., not significant). Conditions include control (no cytokine) and IL‑1β stimulation (1 and 10 ng/ml). Note: Panels are adapted from da Silva et al. (2026), licensed under CC BY 3.0.

## REAGENTS AND SOLUTIONS

### Blocking/Permeabilization buffer

10% (vol/vol) normal donkey serum
1% (wt/vol) BSA
0.1% (vol/vol) Triton X‐100
Prepare in DPBS
Prepare fresh or store up to 1 week at 4°C.

### Collagen type IV stock solution, 2 mg/ml

Dissolve collagen type IV (Sigma‐Aldrich, cat. no. C5533) in 0.25% (vol/vol) acetic acid to 2 mg/ml.

Store in aliquots up to 1 month at 4°C.

### ECM coating solution

100 µg/ml fibronectin
400 µg/ml collagen
Prepare fresh in DPBS on day of use. Do not store. Keep the solution on ice during preparation and use.

### Fibronectin stock solution, 1 mg/ml

Reconstitute the lyophilized human fibronectin (Corning, cat. no. 356008) by adding 5 ml distilled water to the 5 mg vial.

Allow the solution to dissolve for 30 min at room temperature without vortexing or agitation.

Aliquot into single‐use volumes and store at −20°C.

Avoid repeated freeze‐thaw cycles. Use the reconstituted solution within 2 weeks.

### IL‐1β stock solution, 100 µg/ml

Reconstitute recombinant IL‐1β at 100 µg/ml in sterile DPBS containing 0.1% (wt/vol) BSA.

Store in single‐use aliquots up to 3 months at −20°C. Avoid repeated freeze‐thaw cycles.

## Commentary

### Background Information

Chondrocytes are highly responsive to mechanical stimuli, and their phenotype is tightly regulated by the magnitude and duration of applied forces. Among these, FSS associated with synovial fluid movement represents a key regulator of cartilage homeostasis. Physiological levels of FSS promote anabolic activity and ECM synthesis, whereas excessive or sustained mechanical stimulation activates catabolic pathways associated with cartilage degradation and OA progression (Smith et al., 1995; Zhao et al., 2024).

In this article, we provide a detailed, step‐by‐step protocol for reproducibly implementing this platform under optimized experimental conditions. Key parameters, including ECM coating composition and rocker tilt angle, are tuned to promote cell adhesion, viability, and maintenance of a cartilage‐like phenotype.

Under the operating conditions used in this protocol, previous numerical simulations of the platform predicted FSS values ranging from 0.06 to 0.9 Pa across the different microchannel regions (da Silva et al., 2026). As FSS in gravity‐driven systems is inherently time‐dependent, these simulated values correspond to the predicted minimum and maximum shear stresses occurring during the dwell period. Specifically, the maximum FSS (0.9 Pa) was predicted at the bottom surface of Region III immediately after the rocker reached the maximum tilt angle (25°), whereas the minimum FSS (0.06 Pa) was predicted at the bottom surface of Region I at the end of the 300‐s dwell period (da Silva et al., 2026).

Flow characterization provided in Support Protocol 1 is based on experimental measurements of transferred volume and the corresponding flow rate under gravity‐driven rocking conditions. FSS is not directly measured in this protocol. The reported FSS values are based on the numerical characterization of the platform previously described by da Silva et al. (2026), in which time‐dependent COMSOL simulations were performed using the device geometry and operating conditions. Although flow rate and FSS are related, local FSS depends not only on flow rate but also on channel geometry, fluid properties, and boundary conditions. Consequently, the experimentally measured flow rates reported here are used to characterize the gravity‐driven flow behavior of the device, whereas the reported FSS values should be interpreted as estimates derived from the previously published numerical model. Furthermore, biological changes during long‐term culture, including cell attachment and aggregate formation within the microchannels, may alter local hydraulic resistance and, consequently, the mechanical environment experienced by the cells relative to that predicted for the initial channel geometry.

Under optimized conditions, the system supports sustained chondrocyte viability, progressive ECM deposition, and formation of three‐dimensional microtissue‐like aggregates. To model OA‐like conditions, inflammatory stimulation is performed using IL‑1β, which induces a catabolic phenotype characterized by increased secretion of MMPs and pro‐inflammatory cytokines, along with reduced expression of cartilage‐specific ECM components such as collagen type II and aggrecan. Following IL‑1β stimulation, the model reproduces hallmark features of OA, including disruption of tissue architecture and increased catabolic signaling.

Using this protocol, researchers can culture human articular chondrocytes under reproducible gravity‐driven flow conditions, maintain long‐term cartilage‐like phenotypes, and reproducibly induce OA‐like inflammatory responses. The system enables parallel analysis of multiple stimulation conditions within a single experiment and provides a scalable and reproducible platform for studying cartilage mechanobiology under both healthy and inflammatory conditions.

### Critical Parameters

Reproducibility of gravity‐driven microfluidic culture depends on consistent control of surface conditioning, hydrostatic loading, and flow stability. ECM coating composition and concentration are key determinants of initial chondrocyte adhesion and long‐term retention under dynamic culture conditions, with fibronectin and collagen type IV generally supporting stable attachment. Rocker tilt angle is the primary physical parameter governing hydrostatic pressure generation and fluid transport within the microfluidic platform. Consistent angle calibration and stable rocking motion are essential, as flow rate and medium exchange depend directly on these conditions. In this system, a tilt angle of ±10° results in lower flow rates and reduced fluid exchange, whereas ±25° increases perfusion throughout the microfluidic network. All dynamic cultures in this study were performed at ±25° unless otherwise specified. Likewise, maintaining a fixed dwell time (300 sec per tilt) is required to ensure comparable cyclic flow conditions between experiments. Hydrostatic conditions established by reservoir filling configuration (full vs. half‐filled) determine the initial pressure gradient driving flow and must be kept constant across experiments to ensure comparability of flow regimes. Accurate and consistent liquid loading is therefore essential for reproducible hydrodynamic conditions. Flow characterization is performed by measuring the transferred volume and calculating the flow rate. While previous numerical simulations of the platform predicted FSS values ranging from 0.06 to 0.9 Pa (da Silva et al., 2026), these values are not measured directly in the present protocol. Therefore, reproducibility should be evaluated primarily by controlling the flow rate and operating parameters. Proper cell preparation is also critical for experimental consistency. Commercial articular chondrocytes should be maintained as single‐cell suspensions, and standardized thawing density and recovery time should be used to minimize variability in adhesion efficiency and mechanosensitivity. Cell‐counting accuracy directly influences seeding density and thus affects spatial distribution, aggregate formation, and cell‐cell interactions within the microchannels. Finally, IL‐1β concentration and timing (days 15 to 21 in this protocol) define the inflammatory stimulation window and should be applied consistently across experiments to ensure comparable induction of OA‐like phenotypes.

### Troubleshooting

This protocol combines microfluidics, gravity‐driven flow, and cell culture; therefore, most issues arise from loading inconsistencies, surface preparation, or flow instability. Table 1 summarizes the most common problems and their solutions.

**Table 1:** Troubleshooting guide for gravity‐driven microfluidic chondrocyte culture

Problem	Possible cause	Solution
No or weak flow between reservoirs	Incorrect reservoir filling or channel blockage	Verify full/half reservoir configuration; inspect channels before use
Highly variable flow or shear response	Inconsistent loading or rocker settings	Standardize pipetting technique and confirm rocker angle and dwell time
Poor or uneven cell attachment	Insufficient ECM coating or uneven channel filling	Ensure complete channel filling and adequate coating incubation
Cell detachment under flow	Low adhesion strength or insufficient seeding density	Increase initial seeding density or extend static adhesion period
Reduced viability in long‐term culture	Inadequate medium exchange or culture conditions	Replace medium daily and confirm incubator settings
Inconsistent inflammatory response	Degraded or inconsistently applied IL‐1β	Use fresh aliquots and standardize stimulation timing

### Understanding Results

Successful implementation of the microfluidic chondrocyte culture system can be evaluated using a combination of morphological, metabolic, biochemical, and fluorescence‐based readouts, which collectively indicate the formation of cartilage‐like microtissue under controlled gravity‐driven flow conditions. Representative benchmark outcomes are summarized in Fig. 8.

Efficient ECM coating and stable flow conditions are reflected in appropriate chondrocyte adhesion and morphology. Under optimized conditions, fibronectin‐collagen type IV coatings support high cell density and promote a rounded chondrocyte phenotype in regions previously predicted to experience low and intermediate FSS (regions I and II, respectively), while partial elongation may be observed in the region predicted to experience higher FSS levels (region III) (da Silva et al., 2026). In contrast, reduced adhesion, excessive spreading, or cell detachment may indicate suboptimal coating or unstable flow conditions.

Metabolic activity, assessed using the PrestoBlue assay, provides a quantitative measure of culture stability. Under optimal dynamic conditions, metabolic activity is generally maintained or slightly decreases from day 3 to day 21. This behavior is consistent with microfluidic and three‐dimensional culture systems, where chondrocytes adopt a more physiological, less proliferative phenotype (da Silva et al., 2026; Saranya et al., 2023). In contrast, two‐dimensional monolayer cultures often exhibit a progressive increase in metabolic activity due to enhanced proliferation and phenotypic dedifferentiation.

Long‐term culture under dynamic flow promotes progressive ECM deposition and the formation of three‐dimensional microtissue‐like aggregates. These structures become evident by day 14 and further mature by day 21 (Fig. 8A–B). The presence of compact aggregates reflects successful mechanobiological adaptation of chondrocytes to dynamic culture conditions.

Expression of cartilage‐specific ECM markers further confirms maintenance of the chondrocyte phenotype with substantial collagen type II and aggrecan expression observed under control conditions (Fig. 8C). In contrast, reduced expression of both markers is observed following IL‐1β stimulation (Fig. 8C). Previous longitudinal analysis of this microfluidic culture system in the absence of IL‐1β stimulation demonstrated that collagen type II expression increases over time and is most prominent at later stages, particularly in larger microchannel regions, whereas aggrecan expression is typically high at early time points and may decrease gradually while remaining detectable in mature cultures (da Silva et al., 2026). Quantitative analysis (CTCF) shows that IL‐1β stimulation significantly reduces both collagen type II (Fig. 8D) and aggrecan expression (Fig. 8E), indicating ECM degradation.

After establishing a stable baseline, stimulation with IL‐1β induces a characteristic OA‐like phenotype. Cell viability is largely preserved under inflammatory conditions (Fig. 8B), indicating that IL‐1β primarily drives phenotypic alterations rather than cytotoxic effects. However, disruption of microtissue‐like aggregates is observed, consistent with loss of ECM integrity.

Metabolic activity under IL‐1β stimulation may remain stable or increase modestly (Fig. 8F), reflecting a stress‐associated cellular response that may be more pronounced at higher cytokine concentrations.

A robust inflammatory response is further characterized by increased secretion of matrix metalloproteinases (MMP‐1, MMP‐2, MMP‐3, MMP‐7, MMP‐9, and MMP‐13) and pro‐inflammatory cytokines (IL‐1β, IL‐6, and IL‐8), measured in culture supernatants (Fig. 8G–H). Elevated levels relative to control conditions indicate activation of catabolic pathways. Note that the measured IL‐1β concentrations shown in Fig. 8G represent the combined contribution of exogenously added IL‐1β used for inflammatory stimulation (1 ng/ml or 10 ng/ml) and any IL‐1β secreted by the cells during culture. Therefore, IL‐1β measurements should not be interpreted solely as a marker of endogenous cytokine secretion.

In addition, increased levels of collagen type II fragments detected in culture supernatants under IL‐1β stimulation (Fig. 8I) further support ECM degradation. The release of collagen fragments into the medium is consistent with enzymatic breakdown of the cartilage matrix and provides complementary biochemical evidence of the OA‐like phenotype.

Overall, proper execution of the protocol yields reproducible outcomes, including stable chondrocyte adhesion under flow, progressive ECM deposition and microtissue formation, and controlled induction of inflammatory degradation upon IL‐1β stimulation. Deviations such as lack of aggregation, reduced viability, or weak inflammatory responses may indicate issues related to ECM coating, flow calibration, or cytokine exposure conditions.

### Time Considerations

Basic Protocol 1 (microfluidic chip preparation and ECM coating) requires approximately 5 to 6 hr, including coating preparation and incubation steps. Support Protocol 1 (gravity‐driven flow characterization) requires approximately 7 to 8 hr, including coating and equilibration, rocker operation, gravimetric measurements, and data analysis.

Basic Protocol 2 (cell preparation, counting, seeding, and establishment of dynamic culture) requires approximately 1 day for chip seeding and initial static adhesion, followed by long‐term dynamic culture for up to 14 days.

Basic Protocol 3 (IL‐1β‐induced inflammatory stimulation and downstream analysis) requires an additional 7 days following initiation of cytokine treatment on day 15. Endpoint analyses, including fluorescence imaging, metabolic activity assays, immunofluorescence staining, and ELISA measurements, typically require 1 to 10 additional days depending on experimental design and imaging throughput.

The total experimental duration, from chip preparation to completion of inflammatory culture experiments and endpoint analysis, is approximately 21 to 30 days.

### Author Contributions

**Aldeliane M. da Silva**: Conceptualization; methodology; visualization; writing—original draft; writing—review and editing. **Meriem Lamghari**: Methodology; writing—review and editing. **Hoang‐Tuan Nguyen**: Methodology; resources; writing—review and editing. **Jere Kettunen**: Methodology; resources. **Sebastien Mosser**: Conceptualization; methodology; resources; writing—review and editing. **Prateek Singh**: Conceptualization; funding acquisition; methodology; resources; writing—review and editing. **Ali Mobasheri**: Conceptualization; funding acquisition; methodology; writing—review and editing. **Gabriela S. Lorite**: Conceptualization; funding acquisition; methodology; resources; supervision; writing—review and editing. **Priscila Campioni Rodrigues**: Conceptualization; methodology; supervision; writing—original draft; writing—review and editing.

### Conflict of Interest

P. S. and H.‐T. N. declare competing interests as shareholders of Finnadvance Ltd. and Proteins.1.

## Data Availability

No new datasets were generated for this protocol article. The representative data shown in this work were previously published in da Silva et al. (2026) and are available from the original publication (https://doi.org/10.1039/D5LC01167A).

## References

[cpz170489-bib-0001] Carpintero‐Fernández, P. , Varela‐Eirín, M. , García‐Yuste, A. , López‐Díaz, I. , Caeiro, J. R. , & Mayán, M. D. (2022). Osteoarthritis: Mechanistic insights, senescence, and novel therapeutic opportunities. Bioelectricity, 4(1), 39–47. 10.1089/bioe.2021.0039 PMC11441363 39355566

[cpz170489-bib-0002] Conceição, F. , Meneses, J. , Lebre, F. , Becker, M. , Araújo‐Gomes, N. , Vos, R. , Ribeiro, A. R. , Alfaro‐Moreno, E. , Leijten, J. , & Moreira Teixeira, L. (2025). Sex‐stratified osteochondral organ‐on‐chip model reveals sex‐specific responses to inflammatory stimulation. Materials Today Bio, 32, 101728. 10.1016/j.mtbio.2025.101728 PMC12000750 40242482

[cpz170489-bib-0003] da Silva, A. M. , Rodrigues, P. C. , Lamghari, M. , Nguyen, H. T. , Kettunen, J. , Mosser, S. , Singh, P. , Mobasheri, A. , & Lorite, G. S. (2026). High‐throughput microfluidic platform for modelling inflammatory responses of human articular chondrocytes under variable fluid shear stress. Lab Chip, 26(10), 3112–3127. 10.1039/d5lc01167a 41996266

[cpz170489-bib-0004] Dou, H. , Wang, S. , Hu, J. , Song, J. , Zhang, C. , Wang, J. , & Xiao, L. (2023). Osteoarthritis models: From animals to tissue engineering. Journal of Tissue Engineering, 14, 1–23. 10.1177/20417314231172584 PMC10201005 37223125

[cpz170489-bib-0005] Eschweiler, J. , Horn, N. , Rath, B. , Betsch, M. , Baroncini, A. , Tingart, M. , & Migliorini, F. (2021). The biomechanics of cartilage—an overview. Life, 11(4), 302. 10.3390/life11040302 PMC8065530 33915881

[cpz170489-bib-0006] Foster, N. C. , Hall, N. M. , & El Haj, A. J. (2022). Two‐dimensional and three‐dimensional cartilage model platforms for drug evaluation and high‐throughput screening assays. Tissue Engineering Part B: Reviews, 28(2), 421–436. 10.1089/ten.teb.2020.0354 PMC7612674 34010074

[cpz170489-bib-0007] Grenier, S. , Bhargava, M. M. , & Torzilli, P. A. (2014). An in vitro model for the pathological degradation of articular cartilage in osteoarthritis. Journal of Biomechanics, 47(3), 645–652. 10.1016/j.jbiomech.2013.11.050 PMC3938093 24360770

[cpz170489-bib-0008] Hunter, J. D. , March, L. , & Chew, M. (2020). Osteoarthritis in 2020 and beyond: A Lancet Commission. The Lancet, 396(10264), 1711–1712. 10.1016/S0140-6736(20)32230-3 33159851

[cpz170489-bib-0009] Ingber, D. E. (2022). Human organs‐on‐chips for disease modelling, drug development and personalized medicine. Nature Reviews Genetics, 23(8), 467–491. 10.1038/s41576-022-00466-9 PMC8951665 35338360

[cpz170489-bib-0010] Jia, Y. , Le, H. , Wang, X. , Zhang, J. , Liu, Y. , Ding, J. , Zheng, C. , & Chang, F. (2023). Double‐edged role of mechanical stimuli and underlying mechanisms in cartilage tissue engineering. Frontiers in Bioengineering and Biotechnology, 11, 1271762. 10.3389/fbioe.2023.1271762 PMC10694366 38053849

[cpz170489-bib-0011] Lee, D. , Erickson, A. , You, T. , Dudley, A. T. , & Ryu, S. (2018). Pneumatic microfluidic cell compression device for high‐throughput study of chondrocyte mechanobiology. Lab on a Chip, 18(14), 2077–2086. 10.1039/c8lc00320c PMC6467204 29897088

[cpz170489-bib-0012] Liu, H. , Wu, X. , Liu, R. , Wang, W. , Zhang, D. , & Jiang, Q. (2024). Cartilage‐on‐a‐chip with magneto‐mechanical transformation for osteoarthritis recruitment. Bioactive Materials, 33, 61–68. 10.1016/j.bioactmat.2023.10.030 PMC10661690 38024232

[cpz170489-bib-0013] Liu, Q. , Hu, X. , Zhang, X. , Duan, X. , Yang, P. , Zhao, F. , & Ao, Y. (2016). Effects of mechanical stress on chondrocyte phenotype and chondrocyte extracellular matrix expression. Scientific Reports, 6, 37268. 10.1038/srep37268 PMC5112533 27853300

[cpz170489-bib-0014] Manivong, S. , Cullier, A. , Audigié, F. , Banquy, X. , Moldovan, F. , Demoor, M. , & Roullin, V. G. (2023). New trends for osteoarthritis: Biomaterials, models and modeling. Drug Discovery Today, 28(3), 103488. 10.1016/j.drudis.2023.103488 36623796

[cpz170489-bib-0015] Paggi, C. A. , Hendriks, J. , Karperien, M. , & le Gac, S. (2022). Emulating the chondrocyte microenvironment using multi‐directional mechanical stimulation in a cartilage‐on‐chip. Lab on a Chip, 22(9), 1815–1828. 10.1039/d1lc01069g 35352723

[cpz170489-bib-0016] Peitso, V. , Sarmadian, Z. , Henriques, J. , Lauwers, E. , Paggi, C. A. , & Mobasheri, A. (2024). Development of a microphysiological cartilage‐on‐chip platform for dynamic biomechanical stimulation of three‐dimensional encapsulated chondrocytes in agarose hydrogels. Current Protocols, 4(12), e70079. 10.1002/cpz1.70079 PMC11656284 39697087

[cpz170489-bib-0017] Petitjean, N. , Canadas, P. , Royer, P. , Noël, D. , & le Floc'h, S. (2023). Cartilage biomechanics: From the basic facts to the challenges of tissue engineering. Journal of Biomedical Materials Research Part A, 111(7), 1067–1089. 10.1002/jbm.a.37478 36583681

[cpz170489-bib-0018] Saranya, M. , da Silva, A. M. , Karjalainen, H. , Klinkenberg, G. , Schmid, R. , McDonagh, B. , Molesworth, P. P. , Sigfúsdóttir, M. S. , Wågbø, A. M. , Santos, S. G. , Couto, C. , Karjalainen, V. P. , Das Gupta, S. , Järvinen, T. , de Roy, L. , Seitz, A. M. , Finnilä, M. , Saarakkala, S. , Haaparanta, A. M. , … Lorite, G. S. (2023). Magnetic‐responsive carbon nanotubes composite scaffolds for chondrogenic tissue engineering. Advanced Healthcare Materials, 12(30), 2301787. 10.1002/adhm.202301787 PMC11468560 37660271

[cpz170489-bib-0019] Scalzone, A. , Cerqueni, G. , Wang, X. N. , Dalgarno, K. , Mattioli‐Belmonte, M. , Ferreira‐Duarte, A. M. , & Gentile, P. (2023). A cytokine‐induced spheroid‐based in vitro model for studying osteoarthritis pathogenesis. Frontiers in Bioengineering and Biotechnology, 11, 1167623. 10.3389/fbioe.2023.1167623 PMC10203413 37229489

[cpz170489-bib-0020] Smith, R. L. , Donlon, B. S. , Gupta, M. K. , Mohtai, M. , Das, P. , Carter, D. R. , Cooke, J. , Gibbons, G. , Hutchinson, N. , & Schurman, D. J. (1995). Effects of fluid‐induced shear on articular chondrocyte morphology and metabolism in vitro. Journal of Orthopaedic Research, 13(6), 824–831. 10.1002/jor.1100130604 8544017

[cpz170489-bib-0021] Tolabi, H. , Davari, N. , Khajehmohammadi, M. , Malektaj, H. , Nazemi, K. , Vahedi, S. , Ghalandari, B. , Reis, R. L. , Ghorbani, F. , & Oliveira, J. M. (2023). Progress of microfluidic hydrogel‐based scaffolds and organ‐on‐chips for the cartilage tissue engineering. Advanced Materials, 35(26), 2208852. 10.1002/adma.202208852 36633376

[cpz170489-bib-0022] Zhao, J. , Xia, Y. , & He, J. (2024). Low fluid shear stress promotes chondrocyte proliferation and extracellular matrix secretion by downregulating miR‐143‐3p and activating the ERK5/KLF4 signaling pathway. Scientific Reports, 14(1), 27737. 10.1038/s41598-024-78676-w PMC11557884 39532925

